# The potential antidepressant effect of antidiabetic agents: New insights from a pharmacovigilance study based on data from the reporting system databases FAERS and VigiBase

**DOI:** 10.3389/fphar.2023.1128387

**Published:** 2023-02-17

**Authors:** Vera Battini, Robbert P. Van Manen, Michele Gringeri, Giulia Mosini, Greta Guarnieri, Anna Bombelli, Marco Pozzi, Maria Nobile, Sonia Radice, Emilio Clementi, Carla Carnovale

**Affiliations:** ^1^ Unit of Clinical Pharmacology, Department of Biomedical and Clinical Sciences, University Hospital, Università degli Studi di Milano, Milan, Italy; ^2^ Oracle Health Sciences Global Business Unit, Kattendijke, Netherlands; ^3^ Scientific Institute, IRCCS E Medea, Bosisio Parini, Italy

**Keywords:** diabetes, pharmacovigilance, depression, antidiabetic agents, drug repurposing, real-world data, FAERS, VigiBase

## Abstract

**Background:** Growing evidence supports a bidirectional association between diabetes and depression; promising but limited and conflicting data from human studies support the intriguing possibility that antidiabetic agents may be used to relieve effectively depressive symptoms in diabetic patients. We investigated the potential antidepressant effects of antidiabetic drugs in a high-scale population data from the two most important pharmacovigilance databases, *i.e.*, the FDA Adverse Event Reporting System (FAERS) and the VigiBase.

**Material and methods:** From the two primary cohorts of patients treated with antidepressants retrieved from FDA Adverse Event Reporting System and VigiBase we identified *cases* (depressed patients experiencing therapy failure) and *non-cases* (depressed patients experiencing any other adverse event). We then calculated the Reporting Odds Ratio (ROR), Proportional Reporting Ratio (PRR), Empirical Bayes Geometric Mean (EBGM), and Empirical Bayes Regression-Adjusted Mean (ERAM) for *cases versus non-case*s in relation with the concurrent exposure to at least one of the following antidiabetic agent: A10BA Biguanides; A10BB Sulfonylureas; A10BG Thiazolidinediones; A10BH DPP4-inhibitors; A10BJ GLP-1 analogues; A10BK SGLT2 inhibitors (*i.e*., those agents for which preliminary evidence from literature supports our pharmacological hypothesis).

**Results:** For GLP-1 analogues, all the disproportionality scores showed values <1, *i.e.*, statistically significant, in both analyses [from the FAERS: ROR confidence interval of 0.546 (0.450–0.662); PRR (*p*-value) of 0.596 (0.000); EBGM (CI) of 0.488 (0.407–0.582); ERAM (CI) of 0.480 (0.398–0.569) and VigiBase: ROR (CI) of 0.717 (0.559–0.921); PRR (*p*-value) of 0.745 (0.033); EBGM (CI) of 0.586 (0.464–0.733); ERAM of (CI): 0.515 (0.403–0.639)]. Alongside GLP-1 analogues, DPP-4 Inhibitors and Sulfonylureas showed the greatest potential protective effect. With regard to specific antidiabetic agents, liraglutide and gliclazide were associated with a statistically significant decrease in all disproportionality scores, in both analyses.

**Conclusion:** The findings of this study provide encouraging results, albeit preliminary, supporting the need for further clinical research for investigating repurposing of antidiabetic drugs for neuropsychiatric disorders.

## 1 Introduction

Depression, estimated by the World Health Organization (WHO) as the single largest contributor to global disability, is a major challenge for the national health systems. Its co-occurrence with Type 2 Diabetes (T2D) is twice as frequent as might be predicted by chance alone and results in a reduced quality of life and elevated impairment of individuals’ daily functioning (Holt et al., 2014).

A growing number of evidence supports a bidirectional association between diabetes and depression as a result of complex interactions involving brain events and systemic responses (Golden et al., 2008; Laake et al., 2014; Martins et al., 2022). The role of the inflammatory cascade in the induction of metabolic syndrome, oxidative stress and central diseases promoted studies on the identification of novel pharmacological targets for a combined treatment (Lamb and Goldstein, 2008; Chan et al., 2019). The central activation of AMPK, a key enzyme regulating both energy management and psychopathology, which is also supported by some antidiabetic drugs, has been suggested as a useful strategy to relieve both depressive and diabetic symptoms (Pozzi et al., 2019).

Evidence from experimental studies has also reported that traditional anti-hyperglycaemic agents, such as insulin, glyburide, metformin, pioglitazone, vildagliptin, and liraglutide reduce depression-like behaviour in either absence or presence of diabetes (AlHussain et al., 2020; Essmat et al., 2020).

Promising yet still limited clinical evidence from human studies is also available: a recent metanalysis of 9 studies found that GLP1 receptor agonists can relieve depressive symptoms in adult patients affected by T2D (Pozzi et al., 2019) and another study shows that thiazolidinediones might be associated to pharmacologically relevant antidepressant actions (Moulton et al., 2018). However, these concepts still need to be expanded (Odaira et al., 2019).

Antidiabetic agents including metformin, thiazolidinediones GLP-1 agonists and dipeptidyl peptidase 4 (DPP-4) inhibitors are known to cross the blood brain barrier and thus exert both peripheral and central actions. The antidepressant activity of these drugs may be mediated by reducing the blood glucose level, ameliorating the central oxidative stress and inflammation, regulating the hypothalamic–pituitary–adrenal axis, stimulating neuronal growth and protecting from apoptosis through the protein Gs-Protein Kinase A-mediated activation of AMPK (Essmat et al., 2020). The underlying mechanism of action has not been fully elucidated yet.

Spontaneous reporting systems such as the FDA Adverse Event Reporting System (FAERS) and VigiBase represent valuable sources to obtain real-world data on the safety/effectiveness profile of specific drugs, in order to compare therapeutic options, gain insights on potential mechanisms of adverse drug reaction (ADR**)** and (more recently) investigate promising new beneficial effects of drugs, thus contributing to drug repositioning (Cohen et al., 2017; Carnovale et al., 2018; 2019b; 2019a; Mazhar et al., 2019). Due to the insufficient therapeutic response of patients to the available antidepressant medications, drug repositioning may become the most promising strategy to support new indication uses.

Here we report on the antidepressant effect of antidiabetic agents in a high-scale population data from the two largest spontaneous reporting system databases, *i.e*., the FAERS and VigiBase, thus providing new insights in support of their potential drug repurposing in the field of neuropsychiatric disorders.

## 2 Materials and methods

### 2.1 Data source and extraction

This study was designed as a nested case/non-case study. We used the Empirica Signal software (Oracle Health Sciences, Austin, TX) to query the two largest and most comprehensive spontaneous reporting system public databases: the FDA FAERS database (from 1967 up until the end of 2021) and the WHO VigiBase database (from 1968 until the end of September 2021).

Both data sources contain information related to post-marketing safety surveillance reports in the form of Individual Case Safety Reports (ICSRs) submitted by healthcare professionals, consumers, and other sources. Adverse events (AEs) are coded in these two pharmacovigilance databases using the Medical Dictionary for Regulatory Activities (MedDRA^®^) Preferred Terms (PTs) (Fescharek et al., 2004). Each ICSR provides administrative information (country, type of report, qualification of the reporter), patient demographics (sex, age, weight), AEs characteristics (seriousness, date of onset, outcome), details about suspect drug therapy (drug name, exposure start and stop dates, time to onset, dose, route, indication, de-challenge and re-challenge) and information concerning any drug administered at the time of AE but not held responsible for its occurrence by the reporter, referred to as concomitant medication. However, the level of completeness of information varies from case to case (Sakaeda et al., 2013).

Both databases were prepared for data mining, for example by combining initial and follow-up reports into a single case and eliminating obvious duplicate cases using an automated process provided by Oracle.

A primary cohort of ICSRs was defined as all reports mentioning at least one antidepressant drug (ATC Level 3 code N06A “Antidepressants”) as “suspect drug” (either primary or secondary) for any type of AE. Reports containing antidepressants as “concomitant medication” only were not included in the primary cohort.

Within this primary cohort, cases were defined as depressed patients experiencing therapy failure and non-cases as patients experiencing any other AEs. Therapy failure was defined as ICSRs mentioning either the MedDRA narrow SMQ Depression and suicide/self-injury or the narrow SMQ Lack of efficacy (MedDRA version 24.0). (MedDRA-Support Documentation, 2022).

### 2.2 Statistical analysis

By using the Oracle Empirica Signal software (Oracle Health Sciences, Austin, TX), we calculated disproportionality statistics produced by four signal detection methodologies, to assess the occurrence of therapy failure (cases) in depressed patients, in association with the exposure to at least one antidiabetic drug, defined as the following ATC Level 4 codes: A10BA Biguanides; A10BB Sulfonylureas; A10BG Thiazolidinediones; A10BH DPP4-inhibitors; A10BJ GLP-1 analogues; A10BK SGLT2 inhibitors (*i.e*., those agents for which preliminary evidence from literature supports our pharmacological hypothesis).

Three of these disproportionality scores, based on 2 × 2 disproportionality analysis, are well-established and currently used worldwide by several organisations for routine safety surveillance, *i.e*:

i) The Reporting Odds Ratio (ROR), defined as the ratio of the odds of the occurrence of therapy failure with antidiabetic drugs *versus* the occurrence of therapy failure without antidiabetic agents (van Manen et al., 2007);
ii) The Proportional Reporting Ratio (PRR), comparing the frequency of occurrence of therapy failure in reports referring to antidiabetic agents with the frequency of occurrence of reports of therapy failure in reports that do not mention antidiabetic agents. (van Manen et al., 2007).
iii) The Empirical Bayesian Geometric Mean (EBGM) calculated using the Multi-item Gamma Poisson Shrinker (MGPS) Algorithm, using Bayesian shrinkage to improve the reliability of the disproportionality score (DuMouchel, 1999). We generated both the point estimates (EBGM) and their associated 90% confidence intervals labelled EB05–EB95.

Moreover, we used a more advanced regression-based methodology designed to produce disproportionality statistics with adjusted background rates; it can control masking and more extensive confounding effects by fitting separate Bayesian logistic regression models to each target AE and by automatically selecting predictors to be included in each regression model:

iv) The Regression-enhanced Empirical Bayesian Geometric Mean (ERAM) calculated using the Regression-Adjusted Gamma Poisson Shrinker (RGPS) Algorithm (DuMouchel and Harpaz, 2012). We generated the point estimates (ERAM) and their associated 90% confidentiality intervals labelled ER05–ER95.

With the aim to investigate the antidepressant effects of antidiabetic drugs, disproportionality signals were considered clinically meaningful if.

i) The upper limit of the 90% confidence interval (CI) of the ROR for cases (ROR95) is less than one;
ii) The PRR score is less than one and the corresponding *p*-value is less than 0.05;
iii) The upper limit of the 90% confidence interval of the EBGM for cases (EB95) is less than one;
iv) The upper limit of the 90% confidence interval of the ERAM for cases (ER95) is less than one.

## 3 Results

**FIGURE 1:**
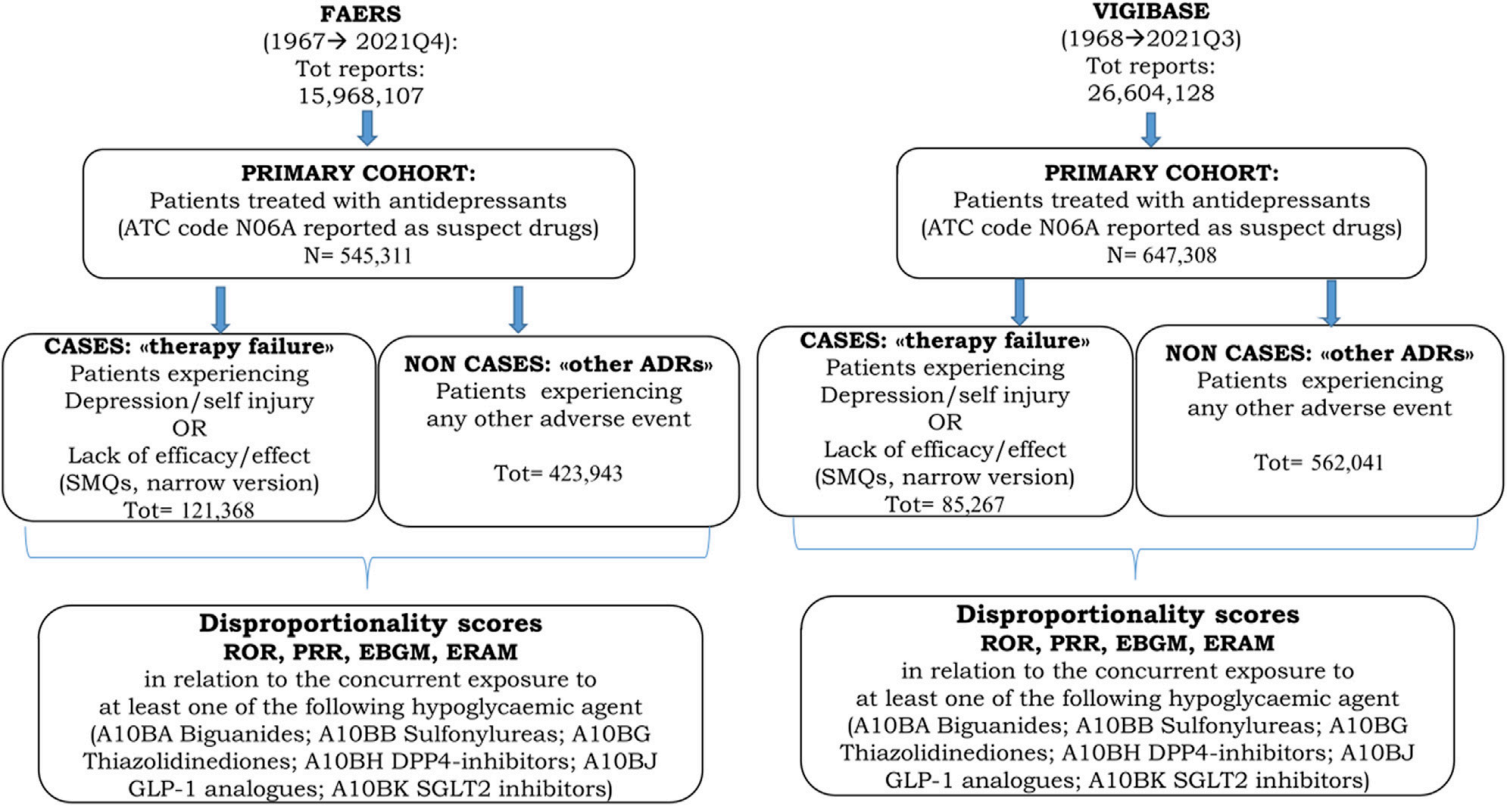
Flow-diagram of data extraction from VigiBase and FAERS.

During the time periods described in the methods, we selected two primary cohorts of ICSRs mentioning antidepressants as “suspect drug” (either primary or secondary) for any AEs reported in the FAERS and VigiBase, which contain 545,311 and 647,308 ICSRs, respectively. Within these primary cohorts we selected 121,368 ICSRs from FAERS and 85,267 from VigiBase as *cases* associated with “therapy failure”; the numbers of *non-cases* for FAERS and VigiBase were 423,943 and 562,041, respectively. Figure 1 shows the flow diagram of data extraction from the two data sources.

Demographical characteristics and type of therapy of depressed patients experiencing therapy failure (*cases*) and other adverse events (*non-cases*) from FAERS and VigiBase are detailed in Tables 1, 2. For *cases*, the most involved age groups reported in the FAERS and VigiBase were 18–44 and 45–74, respectively. In both analyses, >62% of *cases* reported antidepressants as the only suspected drugs and no other drugs. For *non-cases*, the percentage ranged from 41.4% (FAERS) to 80.2% (VigiBase).

**TABLE 1:** Demographical characteristics and therapy of depressed patients (ATC Code N06A) experiencing therapy failure (*cases*) and/or other adverse events (*non-cases*) from the FAERS.

FAERS					
Age Group N° (%) *	Cases (Therapy Failure) (n=121,368)		Non-cases (Other AEs) (n=423,943)		P value
	Age Group years	N° (%)	Age Group years	N° (%)	
	00 - 01	86 (0.07)	00 - 01	6,125 (1.44)	
	02 - 11	928 (0.77)	02 - 11	4,112 (0.97)	
	12 - 17	2,891 (2.38)	12 - 17	5,445 (1.28)	<0.00001
	18 - 44	37,047 (30.52)	18 - 44	110,981 (26.18)	
	45 - 74	34,548 (28.47)	45 - 74	113,167 (26.69)	
	≥75	3,267 (2.69)	≥75	25,191 (5.94)	
	Unknown	42,601 (35.10)	Unknown	158,922 (37.49)	
Gender N° Females (%) *	71,807 (59.16%)		247,489 (58.38%)		<0.00001
Antidiabetic Agents N° (%) *					
*None*	118,585 (97.71%)		409,393 (97.58%)		
*Only one*	1,946 (1.60%)		6,995 (1.67%)		<0.00001
*More than one*	837 (0.72%)		3,156 (0.75%)		
Concomitant insulin, N° (%) *					
Yes	1,085 (0.89%)		5,539 (1.31%)		<0.00001

*χ2-test.

**TABLE 2:** Demographical characteristics and therapy of depressed patients (ATC Code N06A) 601 experiencing therapy failure (*cases*) and/or other adverse events (*non-cases*) from the VigiBase.

VigiBase					
Age Group N° (%) *	Cases (Therapy Failure) (n=85,267)		Non-cases (Other AEs) (n=562,041)		P value
	Age Group years	N° (%)	Age Group years	N° (%)	
	00 - 01	28 (0.04)	00 - 01	4,178 (0.75)	<0.00001
	02 - 11	455 (0.53)	02 - 11	3,240 (0.58)	
	12 - 17	3,172 (3.72)	12 - 17	9,915 (1.76)	
	18 - 44	24,407 (28.62)	18 - 44	152,746 (28.42)	
	45 - 74	26,302 (30.84)	45 - 74	180,889 (32.19)	
	≥75	2,490 (2.92)	≥75	43,277 (7.70)	
	Unknown	28,413 (33.32)	Unknown	160,796 (28.61)	
Gender N° Females (%) *	51,682 (60.61%)		351,353 (62.51%)		<0.00001
Antidiabetic Agents N° (%) *					
*None*	84,405 (98.97%)		559,159 (99.48%)		
*Only one*	649 (0.76%)		1,994 (0.35%)		<0.00001
*More than one*	228 (0.27%)		903 (0.16%)		
Concomitant insulin, N° (%) *					
Yes	192 (0.23%)		784 (0.14%)		<0.00001

*χ2-test.

Supplementary Tables S1, S2 list the number of medications reported as suspect (either primary or secondary suspect) drugs, grouping by ATC Level 2, for *cases* and *non-cases*, in the FAERS and Vigibase.

In both cohorts of depressed patients (*cases* and *non-cases*), more than 58% of individuals were female.

Of depressed subjects experiencing therapy failure, 1,946 and 649 were concomitantly exposed to only one antidiabetic drug, in the FAERS and VigiBase, respectively; in both cohorts, less than 1% was treated with more than one antidiabetic drug and less than 1% was concomitantly exposed to insulin.

Four disproportionality scores (ROR, PRR, EBGM, ERAM) were used to investigate the potential antidepressant effect of antidiabetic drugs. Table 3 shows values for therapy failure in depressed patients exposed to various antidiabetic drug classes.

**TABLE 3:** Disproportionality scores for therapy failure in depressed patients (*cases*) exposed to antidiabetic drug classes.

Data source	Antidiabetic drug class	ROR (ROR05-ROR95)	PRR (*p*-value)	EBGM (EB05-EB95)	ERAM (ER05-ER95)
*FAERS*	*Biguanides*	1.085 (1.038–1.135)	1.068 (0.003)	**0.919 (0.884–0.956)**	**0.856 (0.823–0.890)**
	***Sulfonylureas***	**0.831 (0.776–0.890)**	**0.858 (0.000)**	**0.935 (0.878–0.995)**	**0.858 (0.805–0.912)**
	*Thiazolidinediones*	0.925 (0.810–1.056)	0.938 (0.353)	0.919 (0.815–1.034)	**0.818 (0.723–0.918)**
	***DPP4 Inhibitors***	**0.761 (0.674–0.860)**	**0.796 (0.000)**	**0.687 (0.614–0.767)**	**0.676 (0.602–0.753)**
	***GLP1 Analogues***	**0.546 (0.450–0.662)**	**0.596 (0.000)**	**0.488 (0.407–0.582)**	**0.480 (0.398–0.569)**
	*SGLT2 Inhibitors*	0.901 (0.702–1.158)	0.918 (0.543)	**0.716 (0.571–0.890)**	**0.715 (0.564–0.881)**

Data Source	Drug Class	ROR (ROR05-ROR95)	PRR (*p*-value)	EBGM (EB05-EB95)	ERAM (ER05-ER95)
*VigiBase*	*Biguanides*	1.411 (1.341–1.485)	1.339 (0.000)	1.163 (1.110–1.217)	**0.822 (0.784–0.860)**
	*Sulfonylureas*	**0.830 (0.764–0.902)**	**0.849 (0.000)**	1.010 (0.934–1.092)	**0.838 (0.773–0.904)**
	*Thiazolidinediones*	1.376 (1.184–1.600)	1.311 (0.001)	1.176 (1.025–1.344)	0.926 (0.804–1.055)
	*DPP4 Inhibitors*	0.900 (0.764–1.060)	0.912 (0.312)	**0.840 (0.720–0.974)**	**0.765 (0.653–0.884)**
	***GLP1 Analogues***	**0.717 (0.559–0.921)**	**0.745 (0.033)**	**0.586 (0.464–0.733)**	**0.515 (0.403–0.639)**
	*SGLT2 Inhibitors*	1.401 (1.027–1.911)	1.331 (0.092)	**0.994 (0.755–1.289)**	0.918 (0.685–1.179)

^a^ Disproportionality signals considered clinically meaningful in terms of potential protective effect of antidiabetic drugs are reported in bold.

Among all the drug classes of interest, GLP-1 analogues, DPP-4 Inhibitors and Sulfonylureas showed the greatest potential protective effects. Specifically, all signal detection methodologies and disproportionality statistics investigating the GLP-1 analogues agreed on its potential antidepressant effect and showed values <1, *i.e.*, statistically significant [from the FAERS: ROR (CI) of 0.546 (0.450–0.662); PRR (*p*-value) of 0.596 (0.000); EBGM (CI) of 0.488 (0.407–0.582); ERAM (CI) of 0.480 (0.398–0.569) and VigiBase: ROR (CI) of 0.717 (0.559–0.921); PRR (*p*-value) of 0.745 (0.033); EBGM (CI) of 0.586 (0.464–0.733); ERAM of (CI): 0.515 (0.403–0.639)].

On the other hand, only disproportionality signals in FAERS were considered statistically meaningful for DPP-4 Inhibitors [ROR (CI) of 0.761 (0.674–0.860); PRR (*p*-value) of 0.796 (0.000); EBGM (CI) of 0.687 (0.614–0.767); ERAM (CI) of 0.676 (0.602–0.753)] and Sulfonylureas [ROR (CI) of 0.831 (0.776–0.890); PRR (*p*-value) of 0.858 (0.000); EBGM (CI) of 0.935 (0.878–0.995); ERAM (CI) of 0.858 (0.805–0.912)].

Biguanides, SGLT2 Inhibitors and Thiazolidinediones showed the smallest protective effect. For biguanides we found statistically significant scores only for ERAM in both analyses [FAERS: ERAM (CI) of 0.856 (0.823–0.890); VigiBase: ERAM (CI) of 0.822 (0.784–0.860)]. Similar findings were found for SGLT2 Inhibitors: only the EBGM values were significant in both analyses [FAERS: EBGM (CI) of 0.716 (0.571–0.890)]; VigiBase: EBGM (CI) of 0.994 (0.755–1.289)]. For thiazolidinediones only ERAM from FAERS was statistically significant: ERAM (CI) of 0.818 (0.723–0.918).

In Table 4, detailed disproportionality scores for each antidiabetic drug from both FAERS and VigiBase are reported. With regard to some selected antidiabetic agents, liraglutide and gliclazide were associated in both analyses to a statistically significant decrease in all disproportionality scores. More in detail, in the FAERS analysis, for liraglutide we found the following scores: ROR (CI) of 0.580 (0.438–0.768); PRR (*p*-value) of 0.629 (0.002); EBGM (CI) of 0.534 (0.411–0.683); ERAM (CI) of 0.529 (0.403–0.670)]; consistent results were found for gliclazide: ROR (CI) of 0.527 (0.443–0.628); PRR (*p*-value) of 0.578 (0.000); EBGM (CI) of 0.556 (0.471–0.653); ERAM (CI) of 0.552 (0.465–0.645)]. Findings from VigiBase, considering a larger cohort of patients, supported the previous results; for liraglutide we found: ROR (CI) of 0.519 (0.343–0.785); PRR (*p*-value) of 0.554 (0.011); EBGM (CI) of 0.472 (0.324–0.668); ERAM (CI) of 0.414 (0.275–0.577); for gliclazide we found: ROR (CI) of 0.310 (0.238–0.405); PRR (*p*-value) of 0.341 (0.000); EBGM (CI) of 0.439 (0.340–0.559); ERAM (CI) of 0.438 (0.334–0.553). Supplemental material provides disproportionality scores for *cases* and *non-cases* exposed to antidiabetic drug classes grouping by ATC code level 4 (including details for each antidiabetic drug), in the FAERS (Supplementary Table S3) and VigiBase (Supplementary Table S4).

**TABLE 4:** Disproportionality scores for therapy failure in depressed patients (*cases*) for each antidiabetic drug, from the FAERS and VigiBase analyses.

Drug	ATC code	FAERS (Total cohort: 545,311 depressed subjects)				VigiBase (Total cohort: 647,308 depressed subjects)			
		ROR (ROR05-ROR95)	PRR (*p*-value)	EBGM (EB05-EB95)	ERAM (ER05-ER95)	ROR (ROR05-ROR95)	PRR (*p*-value)	EBGM (EB05-EB95)	ERAM (ER05-ER95)
Canagliflozin	SGLT2-inhibitors	1.268 (0.826–1.945)	1.208 (0.439)	0.952 (0.662–1.335)	0.894 (0.605–1.229)	2.797 (1.655–4.726)	2.262 (0.002)	1.466 (0.977–2.131)	1.169 (0.746–1.670)
**Chlorpropamide**	**Sulfonylureas**	**0.253** (**0.119–0.539)**	**0.294** (**0.002)**	0.819 (0.441–1.416)	0.767 (0.382–1.260)	0.092 (0.017–0.480)	**0.104 (0.005)**	0.888 (0.393–1.784)	0.715 (0.247–1.380)
**Dapagliflozin**	**SGLT2-inhibitors**	**0.543** (**0.304–0.971)**	0.593 (0.106)	**0.565 (0.343–0.888)**	**0.573 (0.331–0.870)**	0.791 (0.389–1.610)	0.813 (0.729)	0.714 (0.410–1.175)	0.659 (0.345–1.055)
**Dulaglutide**	**GLP-1 analogues**	**0.373** (**0.217–0.641)**	**0.422** (**0.003)**	**0.375 (0.232–0.580)**	**0.382 (0.226–0.571)**	0.722 (0.391–1.333)	0.750 (0.476)	0.668 (0.403–1.053)	**0.629** (**0.354–0.969)**
Empagliflozin	SGLT2-inhibitors	0.911 (0.595–1.395)	0.926 (0.815)	0.721 (0.496–1.018)	**0.723 (0.485–1.001**	1.279 (0.777–2.106)	1.234 (0.517)	0.966 (0.636–1.419)	0.945 (0.593–1.363)
Exenatide	GLP-1 analogues	0.791 (0.571–1.096)	0.823 (0.274)	**0.711 (0.529–0.938)**	**0.649 (0.475–0.845)**	1.106 (0.770–1.590)	1.091 (0.731)	0.864 (0.626–1.169)	**0.722** (**0.510–0.964)**
**Gliclazide**	**Sulfonylureas**	**0.527** (**0.443–0.628)**	**0.578** (**0.000)**	**0.556 (0.471–0.653)**	**0.552 (0.465–0.645)**	**0.310** (**0.238–0.405)**	**0.341 (0.000)**	**0.439** (**0.340–0.559)**	**0.438** (**0.334–0.553)**
Glimepiride	Sulfonylureas	0.937 (0.808–1.087)	0.948 (0.500)	0.918 (0.802–1.047)	**0.853 (0.742–0.970)**	0.984 (0.833-1–162)	0.986 (0.912)	0.969 (0.830–1.126)	**0.851** (**0.726–0.985)**
Glipizide	Sulfonylureas	1.123 (0.987–1.277)	1.098 (0.149)	1.186 (1.057–1.327)	1.043 (0.927–1.164)	1.502 (1.304–1.728)	1.409 (0.000)	1.431 (1.260–1.619)	1.087 (0.954–1.228)
Gliquidone	Sulfonylureas	0.440 (0.078–2.472)	0.491 (0.677)	0.864 (0.352–1.844)	0.806 (0.275–1.563)	0.942 (0.162–5.466)	0.949 (0.641)	1.185 (0.527–2.375)	1.026 (0.355–1.980)
Linagliptin	Dpp-4 Inhibitors	0.669 (0.446–1.003)	0.713 (0.125)	**0.651 (0.452–0.913)**	**0.654 (0.443–0.900)**	0.642 (0.382–1.079)	0.673 (0.199)	0.720 (0.460–1.084)	**0.645** (**0.390–0.951)**
**Liraglutide**	**GLP-1 analogues**	**0.580** (**0.438–0.768)**	**0.629** (**0.002)**	**0.534 (0.411–0.683)**	**0.529 (0.403–0.670)**	**0.519** (**0.343–0.785)**	**0.554 (0.011)**	**0.472** (**0.324–0.668)**	**0.414** (**0.275–0.577)**
Lixisenatide	GLP-1 analogues	0.629 (0.108–3.651)	0.675 (0.986)	0.871 (0.355–1.858)	**0.883 (0.302–1.712)**	2.197 (0.329–14.683)	1.898 (0.968)	1.160 (0.516–2.326)	1.089 (0.377–2.102)
Metformin	Biguanides	1.107 (1.057–1.159)	1.085 (0.000)	**0.933 (0.896–0.972)**	**0.866 (0.831–0.901)**	1.420 (1.349–1.494)	1.345 (0.000)	1.166 (1.113–1.220)	1.200 (1.145–1.255)
Pioglitazone	Thiazolidinediones	1.030 (0.869–1.220)	1.024 (0.816)	0.934 (0.802–1.084)	**0.821 (0.701–0.948)**	1.382 (1.138–1.678)	1.316 (0.007)	1.121 (0.940–1.328)	0.883 (0.736–1.042)
Rosiglitazone	Thiazolidinediones	0.909 (0.717–1.152)	0.924 (0.553)	0.923 (0.744–1.134)	**0.815 (0.651–0.993)**	1.378 (1.070–1.775)	1,313 (0.045)	1.187 (0.946–1.475)	0.895 (0.705–1.104)
**Saxagliptin**	**Dpp-4 Inhibitors**	**0.372** (**0.185–0.749)**	**0.421** (**0.023)**	**0.485 (0.271–0.815)**	**0.488 (0.254–0.782)**	0.638 (0.316–1.289)	0.670 (0.378)	0.706 (0.405–1.161)	0.643 (0.336–1.029)
**Semaglutide**	**GLP-1 analogues**	**0.267** (**0.114–0.622)**	**0.309** (**0.009)**	**0.349 (0.178–0.626)**	**0.330 (0.155–0.558)**	-	-	-	**-**
Sitagliptin	Dpp-4 Inhibitors	0.968 (0.830–1.130)	0.974 (0.767)	**0.840 (0.730–0.962)**	**0.809 (0.701–0.924)**	1.119 (0.929–1.347)	1.102 (0.348)	0.991 (0.836–1.169)	0.863 (0.723–1.014)
Tolazamide	Sulfonylureas	0.315 (0.057–1.726)	0.360 (0.396)	0.986 (0.403–2.103)	0.877 (0.300–1.701)	0.824 (0.144–4.717)	0.844 (0.757)	1.239 (0.551–2.484)	1.073 (0.371–2.071)
**Troglitazone**	**Thiazolidinediones**	**0.318 (0.159–0.638)**	**0.364** (**0.006)**	0.808 (0.452–1.357)	0.717 (0.374–1.151)	0.507 (0.189–1.359)	0.542 (0.354)	1.087 (0.555–1.962)	0.985 (0.432–1.721)
**Vildagliptin**	**Dpp-4 Inhibitors**	**0.304** (**0.152–0.608)**	**0.349** (**0.004)**	**0.421 (0.235–0.707)**	**0.458 (0.239–0.734)**	**0.449** (**0.224–0.900)**	0.485 (0.073)	0.631 (0.362–1.038)	0.676 (0.354–1.082)

^a^ Disproportionality signals considered clinically meaningful in terms of potential protective effect of antidiabetic drugs are reported in bold.

## 4 Discussion

Studies on glucose-lowering agents may have a positive influence on the symptoms of depression, although the evidence from animal and human studies is scarce and conflicting (Monnier et al., 2006; Ceriello et al., 2013; Fiorentino et al., 2013).

This is the first study aimed at evaluating the potential antidepressant effect of antidiabetic agents in a high-scale population data (we included two cohorts of 121,368 and 85,267 depressed patients experiencing therapy failure) from the two largest spontaneous reporting system databases, *i.e.*, the FAERS and VigiBase, thus providing new insights for improving the knowledge on this topic and supporting the need for further research on antidiabetic drug repurposing in the field of neuropsychiatric disorders.

It is well-known that pharmacovigilance databases were originally intended to track frequent adverse events; however, when a sufficient amount of data is available, they can also be used to indirectly track the beneficial outcomes through monitoring reductions of related adverse event frequencies (Cohen et al., 2017).

From this perspective, reported adverse drug events may serve as useful indicators to predict new opportunities for drug repositioning, making spontaneous reporting system databases valuable data sources for driving further research in the discover of new and effective uses of drugs (Pushpakom et al., 2019).

Overall, the investigated antidiabetic drug classes showed a beneficial effect to depressed patients, albeit with a high heterogeneity in terms of statistically significant decrease in disproportionality scores, thus suggesting that some specific pharmacological agents may exert a more prominent beneficial effect.

In our study, GLP-1 analogues showed the greatest potential protective effect in the cohort of depressed patients experiencing therapy failure that we analysed. Of importance, all signal-detection methodologies and disproportionality statistics we used to investigate the antidepressant effect of GLP-1 analogues showed values statistically significant (<1) in both pharmacovigilance databases (Table 3), with a ROR ranging from 0.546 (0.450–0.662) to 0.717 (0.559–0.921), in the FAERS and VigiBase, respectively. ROR is the most used disproportionality score worldwide for routine safety surveillance.

However, more recently, many Authors applied this approach to the FAERS and VigiBase to identify candidates for drug repositioning in a variety of clinical research areas (e.g., psychiatry, neurology, cardiology), by searching for an inverse signal, postulating that drugs that demonstrated an under-reporting of AEs of interest could be protective against these AEs (Wang et al., 2016; Horinouchi et al., 2018; Hosomi et al., 2018; Chrétien et al., 2021).

To test our hypothesis, we have expanded this approach further by also providing also other well-established scores based on 2 × 2 disproportionality analysis (PRR and EBGM), and a more advanced regression-based methodology designed to produce disproportionality statistics with adjusted background rates: it can control for masking and more extensive confounding effects by fitting separate Bayesian logistic regression models to each target AE and by automatically selecting predictors to be included in each regression model, i.e., ERAM (DuMouchel and Harpaz, 2012).

In both pharmacovigilance databases, ERAM values suggest GLP-1 analogues may exert a clinical meaningful protective effect, as demonstrated by significant reductions of depression-like symptom frequencies in patients with depression and diabetes [point estimates: 0.480 (0.398–0.569) in FAERS and 0.515 (0.403–0.639), in VigiBase].

When focusing on specific drugs, liraglutide was associated with a statistically significant decrease in all disproportionality scores. Data from the FAERS-based study [ROR (CI) of 0.580 (0.438–0.768); PRR (*p*-value) of 0.629 (0.002); EBGM (CI) of 0.534 (0.411–0.683); ERAM (CI) of 0.529 (0.403–0.670)], support the hypothesis that this antidiabetic agent might exert beneficial effects to depressed patients. Interestingly, when investigating the potential protective effect of liraglutide in a larger cohort of patients, findings from VigiBase strongly supported the previous results [ROR (CI) of 0.519 (0.343–0.785); PRR (*p*-value) of 0.554 (0.011); EBGM (CI) of 0.472 (0.324–0.668); ERAM (CI) of 0.414 (0.275–0.577)].

In line with our findings, clinical and preclinical studies, albeit very scant, support these encouraging results.

Clinical trials have demonstrated improvements in anhaedonia in patients treated with liraglutide (Mansur et al., 2017). The administration of this drug in diabetic mice has demonstrated neuroprotective (Porter et al., 2012; Li et al., 2015; Gumuslu et al., 2016), anxiolytic and anti-depressant effects in a Type 1 Diabetes (T1D) rat model. The drug was also found to increase neurogenesis in the mouse brain (Hunter and Hölscher, 2012) and to enhance effects on synaptic plasticity (McClean et al., 2010).

It has been postulated that, incretins might exert neuropsychiatric effects given the presence of GLP-1 receptors in the central nervous system; stimulation of GLP-1 receptors has shown effects on mitochondrial functions, neuroinflammation, synaptic plasticity, learning and memory, serotonin turnover, serotonin-receptor expression in the amygdala and central dopamine levels, in multiple experimental models of both neurological diseases and depression (athauda and Foltynie, 2016; Athauda and Foltynie, 2016; Kim et al., 2020; van Bloemendaal et al., 2014).

Our study shows that also DPP-4 Inhibitors show potential anti-depressant activity, as supported by all significant values of the disproportionality scores from the FAERS; however, when expanding analysis in a larger cohort of patients (*i.e.,* in VigiBase), only EBGM and ERAM scores were of meaningful clinical relevance, suggesting an important but less prominent antidepressant effect compared to GLP-1 analogues. Within this latter drug class, saxagliptin and vildagliptin showed a significant reductions of depression-like symptom frequencies in patients with depression and diabetes, in all analyses we carried out in the FAERS (*i.e.*, values from all disproportionality scores are significant) (see Table 4), adding preliminary and encouraging evidence (but less promising than those reported for GLP-1 analogues) to the very limited existing body of knowledge on the potential use of DPP-4 Inhibitors as an adjuvant in the treatment of depression. Recent data show that sitagliptin has mild anti-depressant effect in a depression model (Kamble et al., 2016). and a better antidepressant activity than imipramine (Saritha and Chandrashekar, 2018). However, a recent randomised controlled trial (RCT) did not detect evidence of superiority of sitagliptin over placebo for depressive symptoms in 44 patients with T2D, possibly due to the small sample size and limited treatment duration (Moulton et al., 2021). To address the issue further, an on-going randomised double-blind trial including 80 adult outpatients with major depression is evaluating the antidepressant effects of vildagliptin 50 mg *versus* escitalopram 20 mg (ClinicalTrial.gov, 2022).

In line with the overall picture regarding DPP-4 Inhibitors, sulfonylureas showed a similar potential: All values of the disproportionality scores from the FAERS and 3 out of 4 from VigiBase were of significant importance. These preliminary results may serve as indicators for supporting further research to better investigate their beneficial effects to depressed patients as the currently available evidence is scant and relatively conflicting. A recent experimental study showed that the glyburide exerts an effect on modulating depressive like-behaviour together with insulin resistance *via* an NLRP3-inflammasome inhibition (Su et al., 2017). Indeed, NLRP3 may be involved in the pathophysiology of depression (Alcocer-Gómez et al., 2014; 2016), supporting its role as promising therapeutic target for depression.

A population-based cohort study found that sulfonylureas in combination with metformin decrease the risk of affective disorders in T2D patients (Wahlqvist et al., 2012). In contrast, high doses of sulfonylureas were associated with higher risk of depression in a recent population-based cohort and nested case-control study (Wium-Andersen et al., 2022).

We found that biguanides, SGLT2 inhibitors and thiazolidinediones are associated to antidepressant beneficial effects, albeit the entity of this effect is not statistically significant for all disproportionality scores, neither in the FAERS nor in VigiBase.

Among the above-mentioned drug classes, metformin is one of the most investigated antidiabetic drugs as potential adjuvant therapy in depressed patients. Empirical insights showed that it ameliorates stress-induced depression-like behaviours through the enhancement of BDNF expression *via* AMPK/CREB-mediated histone acetylation (Fang et al., 2020) and it has been shown to elicit marked anti-inflammatory, antioxidant, and neuroprotective activities and to improve memory and learning functions in rats (Pintana et al., 2012; Shivavedi et al., 2017).

Recently, in a case–control study, metformin was a protective factor against depression in elderly diabetic patients, as suggested by the adjusted OR of 0.567 (95% CI: 0.323–0.997; *p* < 0.05) (Chen et al., 2019). In older men with T2D and high frailty risk, metformin was associated with a 15.6% decrease in depression (Wang et al., 2017). In our FAERS analysis, among biguanides, metformin was associated with the lowest occurrence of depression-like symptoms compared to non-users of this medication, as confirmed by the two statistically significant disproportionality scores EBGM [0.933 (0.896–0.972)] and ERAM [0.866 (0.831–0.901)], based on Bayesian statistical methods and regression-based methodology, respectively.

As a consequence of the high heterogeneity of previous studies in terms of methodological approaches, it is not possible to directly compare data from different scores; however, our findings support all these previous encouraging results. On the other hand, it is worth mentioning that a recent meta-analysis of clinical trials failed to find an effect of metformin on depression risk, while suggesting a potential role of pioglitazone (Moulton et al., 2018).

Among SGLT2 inhibitors, dapagliflozin was the drug associated with the lowest occurrence of depression-like symptoms compared to non-users of this drug, as confirmed by three statistically significant disproportionality scores ROR [0.543 (0.304–0.971)], EBGM [0.565 (0.343–0.888)] and ERAM [0.573 (0.331–0.870)], from our FAERS analysis. To date, positive but very limited evidence both on their potential neuroprotective effect and the likely underlying mechanism was available for SGLT2 inhibitors (Şahin et al., 2020). Studies have highlighted their antioxidant, anti-inflammatory, and antiapoptotic mechanisms, regardless of their glycaemic control benefits (Shaikh et al., 2016; Sa-Nguanmoo et al., 2017; El-Sahar et al., 2020; Esterline et al., 2020; Wiciński et al., 2020; Muhammad et al., 2021). Dapaglifozin attenuated depressive-like behaviour of male rats in the forced swim test and was also found to be comparable to imipramine in the treatment of mild-to-moderate depression (Cam et al., 2019). In humans, these drugs improved the quality of life of people with diabetes (maybe due to the weight loss observed in the enrolled patients); however, no change in terms of Pittsburgh Sleep Quality, and Beck Anxiety Inventory scores was found.

### 4.1 Strengths and limitations

This is the first study aimed at providing a comprehensive overview of the potential beneficial antidepressant effect of antidiabetic agents in a high-scale population data from the two largest spontaneous reporting system databases, *i.e*., FAERS and VigiBase.

Pharmacovigilance databases are commonly used to track frequent adverse events; however, with a sufficient amount of data, they may also be used to investigate the beneficial outcomes through monitoring reductions in frequency of the related adverse events (Cohen et al., 2017).

The spontaneously reported adverse drug events may serve as useful indicators to predict new opportunities for drug repositioning, making spontaneous reporting system databases valuable data sources for driving clinical research (Pushpakom et al., 2019).

Growing number of evidence supports this innovative approach based on the use of pharmacovigilance databases, especially FAERS and VigiBase, to investigate promising new beneficial effects of drugs in real-world clinical practice, in a variety of clinical settings (e.g., psychiatry, neurology, cardiology) (Wang et al., 2016; Cohen et al., 2017; Carnovale et al., 2018; 2019b; 2019a; Horinouchi et al., 2018; Hosomi et al., 2018; Mazhar et al., 2019; Chrétien et al., 2021).

Furthermore, as real-world data (RWD), including spontaneously reported adverse events, refer to a large amount of clinical data collected during the patient’s daily life, they can address intrinsic limitations of traditional clinical trials, such as highly selected populations, strict inclusion/exclusion criteria, small sample sizes, short follow-up periods, with consequent lack of external validity.

Indeed, RWD are often used to focus on special populations who are usually excluded from RCTs, such as patients receiving polytherapy, children, pregnant women, and elderly people (Trifirò et al., 2019); RWD are hence gaining increasing attention in the whole drug life-cycle process, including regulatory decision-making. In our study, the large set of data (we included two cohorts of 121,368 and 85,267 depressed patients experiencing therapy failure, from FAERS and VigiBase, respectively) provided sufficient statistical power for the analysis to generate hypotheses for unknown potential uses.

On the other hand, it is well known that the use of pharmacovigilance databases has some intrinsic limitations: reporting might be influenced by factors such as notoriety bias, selection bias and under-reporting and there is no certainty that the reported event was causally related due to the suspect drug. Moreover, as these data sources are designed to report adverse events, unintentional beneficial effects of the drug therapy could not be recorded. Pharmacovigilance data cannot be eventually used to calculate the incidence rates of events. In view of the above-mentioned limitations of pharmacovigilance databases, it is worth mentioning that RCTs are non-etheless the gold standard in evidence-based medicine for demonstrating drug efficacy (Compher, 2010) and new clinical studies specifically designed at investigating the role of antidiabetics in depressed patients are needed.

## 5 Conclusion

All the antidiabetic drug classes investigated in our pharmacoepidemiological study showed a potential beneficial effect to depressed patients (in terms of a decreased occurrence of therapy failure/depression-related symptoms), with a high heterogeneity in terms of statistically significant disproportionality scores. This comprehensive overview suggests that some specific pharmacological agents, in particular, GLP-1 analogues might exert a more prominent beneficial and clinically meaningful effects. Due to the insufficient therapeutic response of patients to the available antidepressant medications, repositioning of antidiabetic drugs might become a valuable new approach to improve drug treatment in depression. In view of the nature of this study, the result of this research is not an ultimate conclusion, but a suggestion for further clinical research. Gold-standard RCTs are warranted to confirm these encouraging results, albeit preliminary, and properly characterize the topic.

## Data Availability

The original contributions presented in the study are included in the article/Supplementary Material, further inquiries can be directed to the corresponding author.
