# Supplementary material for: The potential antidepressant effect of antidiabetic agents: New insights from a pharmacovigilance study based on data from the reporting system databases FAERS and VigiBase
Source: Front Pharmacol. 2023 Feb 17;14:1128387. doi: 10.3389/fphar.2023.1128387 (PMC9981969; doi:10.3389/fphar.2023.1128387)
Supplement: Supplementary file 1 [file Table1.DOCX]

**STable 1.** Number of medications reported as suspect (either primary or secondary suspect) drugs, grouping by ATC Level 2, for *cases* (a) and *non-cases* (b), in the FAERS.

| **a) *CASES*** | | |
| --- | --- | --- |
| **ATC Level 2** | **Drug references** | |
| Psychoanaleptics | 196163 | |
| Psycholeptics | 70847 | |
| Antiepileptics | 29430 | |
| Analgesics | 24894 | |
| Drugs For Acid Related Disorders | 9263 | |
| Antiinflammatory And Antirheumatic Products | 8906 | |
| Drugs For Obstructive Airway Diseases | 8180 | |
| Antihistamines For Systemic Use | 7916 | |
| Vitamins | 7774 | |
| Beta Blocking Agents | 7308 | |
| Agents Acting On The Renin-Angiotensin System | 6701 | |
| Lipid Modifying Agents | 6100 | |
| Cough And Cold Preparations | 5743 | |
| Drugs Used In Diabetes | 5613 | |
| Muscle Relaxants | 5527 | |
| Anesthetics | 5277 | |
| Calcium Channel Blockers | 5178 | |
| Sex Hormones And Modulators Of The Genital System | 5108 | |
| Other Nervous System Drugs | 5042 | |
| Thyroid Therapy | 4851 | |
| Diuretics | 4807 | |
| Antibacterials For Systemic Use | 4476 | |
| Immunosuppressants | 4260 | |
| Antiseptics And Disinfectants | 4100 | |
| Unknown [V11] | 3660 | |
| Stomatological Preparations | 3232 | |
| Anti-Parkinson Drugs | 2715 | |
| Unclassified | 2674 | |
| Antidiarrheals, Intestinal Antiinflammatory/Antiinfective Agents | 2485 | |
| Mineral Supplements | 2231 | |
| Antineoplastic Agents | 2218 | |
| Antithrombotic Agents | 2153 | |
| Urologicals | 2085 | |
| Antihypertensives | 2025 | |
| Antivirals For Systemic Use | 1955 | |
| Cardiac Therapy | 1894 | |
| Drugs For Functional Gastrointestinal Disorders | 1592 | |
| Drugs For Constipation | 1558 | |
| Corticosteroids For Systemic Use | 1414 | |
| Blood Substitutes And Perfusion Solutions | 1174 | |
| General Nutrients | 1164 | |
| Nasal Preparations | 997 | |
| Antiemetics And Antinauseants | 955 | |
| Antianemic Preparations | 918 | |
| Drugs For Treatment Of Bone Diseases | 762 | |
| Antiprotozoals | 727 | |
| Gynecological Antiinfectives And Antiseptics | 639 | |
| All Other Therapeutic Products | 606 | |
| Antiobesity Preparations, Excl. Diet Products | 565 | |
| Unspecified Herbal | 543 | |
| Ophthalmologicals | 526 | |
| Immunostimulants | 516 | |
| Endocrine Therapy | 458 | |
| Antigout Preparations | 455 | |
| Corticosteroids, Dermatological Preparations | 436 | |
| Antifungals For Dermatological Use | 344 | |
| Antimycotics For Systemic Use | 334 | |
| Anti-Acne Preparations | 231 | |
| Other Gynecologicals | 224 | |
| Pituitary And Hypothalamic Hormones And Analogues | 222 | |
| Vaccines | 185 | |
| Vasoprotectives | 183 | |
| Antimycobacterials | 171 | |
| Calcium Homeostasis | 169 | |
| Other Dermatological Preparations | 148 | |
| Bile And Liver Therapy | 146 | |
| Digestives, Incl. Enzymes | 131 | |
| Antipsoriatics | 127 | |
| Other Alimentary Tract And Metabolism Products | 121 | |
| Diagnostic Agents | 119 | |
| Antihemorrhagics | 106 | |
| Immune Sera And Immunoglobulins | 84 | |
| Peripheral Vasodilators | 79 | |
| Unknown [H11] | 79 | |
| Topical Products For Joint And Muscular Pain | 77 | |
| Anthelmintics | 70 | |
| Antipruritics, Incl. Antihistamines, Anesthetics, Etc. | 61 | |
| Antibiotics And Chemotherapeutics For Dermatological Use | 56 | |
| Other Respiratory System Products | 52 | |
| Ectoparasiticides, Incl. Scabicides, Insecticides And Repellents | 47 | |
| Pancreatic Hormones | 43 | |
| Unknown [C11] | 23 | |
| Other Drugs For Disorders Of The Musculo-Skeletal System | 22 | |
| Contrast Media | 21 | |
| All Other Non-Therapeutic Products | 18 | |
| Preparations For Treatment Of Wounds And Ulcers | 13 | |
| Emollients And Protectives | 12 | |
| Unknown [A21] | 12 | |
| Anabolic Agents For Systemic Use | 11 | |
| Diagnostic Radiopharmaceuticals | 10 | |
| Other Hematological Agents | 9 | |
| Throat Preparations | 8 | |
| Otologicals | 7 | |
| Therapeutic Radiopharmaceuticals | 7 | |
| Tonics | 5 | |
| Allergens | 1 | |
| Medicated Dressings | 1 | |
| Unknown [D12] | 1 | |
| Unknown [N11] | 1 | |
| Unknown [N999] | 1 | |
| ***b) NON-CASES*** |  |  |
| **ATC Level 2** | **Drug references** |  |
| Psychoanaleptics | 570034 |  |
| Psycholeptics | 169643 |  |
| Analgesics | 66528 |  |
| Antiepileptics | 60996 |  |
| Drugs For Acid Related Disorders | 41336 |  |
| Vitamins | 32845 |  |
| Antibacterials For Systemic Use | 29174 |  |
| Drugs For Obstructive Airway Diseases | 28139 |  |
| Agents Acting On The Renin-Angiotensin System | 26901 |  |
| Diuretics | 26317 |  |
| Beta Blocking Agents | 24747 |  |
| Lipid Modifying Agents | 24489 |  |
| Drugs Used In Diabetes | 22638 |  |
| Antiinflammatory And Antirheumatic Products | 22511 |  |
| Antihistamines For Systemic Use | 21116 |  |
| Sex Hormones And Modulators Of The Genital System | 20806 |  |
| Thyroid Therapy | 18853 |  |
| Other Nervous System Drugs | 17211 |  |
| Calcium Channel Blockers | 16364 |  |
| Cough And Cold Preparations | 15820 |  |
| Antithrombotic Agents | 15541 |  |
| Stomatological Preparations | 15355 |  |
| Anesthetics | 13389 |  |
| Unknown [V11] | 11732 |  |
| Muscle Relaxants | 11652 |  |
| Cardiac Therapy | 11374 |  |
| Anti-Parkinson Drugs | 10251 |  |
| Drugs For Constipation | 9352 |  |
| Unclassified | 8660 |  |
| Immunosuppressants | 8406 |  |
| Urologicals | 8301 |  |
| Mineral Supplements | 8067 |  |
| Antidiarrheals, Intestinal Antiinflammatory/Antiinfective Agents | 7705 |  |
| Blood Substitutes And Perfusion Solutions | 7606 |  |
| Drugs For Functional Gastrointestinal Disorders | 7575 |  |
| Antineoplastic Agents | 7456 |  |
| Antihypertensives | 6280 |  |
| Antiseptics And Disinfectants | 5850 |  |
| Antivirals For Systemic Use | 5489 |  |
| Corticosteroids For Systemic Use | 5355 |  |
| Antianemic Preparations | 5049 |  |
| Nasal Preparations | 3851 |  |
| General Nutrients | 3734 |  |
| Antiemetics And Antinauseants | 3724 |  |
| Drugs For Treatment Of Bone Diseases | 3020 |  |
| Antigout Preparations | 2739 |  |
| All Other Therapeutic Products | 2622 |  |
| Antiobesity Preparations, Excl. Diet Products | 2572 |  |
| Ophthalmologicals | 2407 |  |
| Gynecological Antiinfectives And Antiseptics | 2381 |  |
| Unspecified Herbal | 2191 |  |
| Antiprotozoals | 2096 |  |
| Endocrine Therapy | 1907 |  |
| Antimycotics For Systemic Use | 1890 |  |
| Immunostimulants | 1477 |  |
| Antifungals For Dermatological Use | 1330 |  |
| Corticosteroids, Dermatological Preparations | 1301 |  |
| Vasoprotectives | 832 |  |
| Other Gynecologicals | 806 |  |
| Digestives, Incl. Enzymes | 794 |  |
| Other Alimentary Tract And Metabolism Products | 773 |  |
| Antimycobacterials | 767 |  |
| Vaccines | 749 |  |
| Bile And Liver Therapy | 748 |  |
| Peripheral Vasodilators | 733 |  |
| Pituitary And Hypothalamic Hormones And Analogues | 643 |  |
| Calcium Homeostasis | 632 |  |
| Anti-Acne Preparations | 550 |  |
| Antihemorrhagics | 515 |  |
| Other Dermatological Preparations | 456 |  |
| Immune Sera And Immunoglobulins | 390 |  |
| Unknown [H11] | 304 |  |
| Other Drugs For Disorders Of The Musculo-Skeletal System | 299 |  |
| Antipsoriatics | 272 |  |
| Antibiotics And Chemotherapeutics For Dermatological Use | 258 |  |
| Topical Products For Joint And Muscular Pain | 242 |  |
| Ectoparasiticides, Incl. Scabicides, Insecticides And Repellents | 185 |  |
| All Other Non-Therapeutic Products | 177 |  |
| Diagnostic Radiopharmaceuticals | 162 |  |
| Diagnostic Agents | 151 |  |
| Antipruritics, Incl. Antihistamines, Anesthetics, Etc. | 145 |  |
| Contrast Media | 119 |  |
| Emollients And Protectives | 102 |  |
| Anthelmintics | 97 |  |
| Unknown [C11] | 90 |  |
| Unknown [A21] | 87 |  |
| Anabolic Agents For Systemic Use | 84 |  |
| Other Respiratory System Products | 66 |  |
| Pancreatic Hormones | 53 |  |
| Preparations For Treatment Of Wounds And Ulcers | 52 |  |
| Therapeutic Radiopharmaceuticals | 41 |  |
| Other Hematological Agents | 29 |  |
| Otologicals | 20 |  |
| Allergens | 16 |  |
| Throat Preparations | 13 |  |
| Tonics | 12 |  |
| Investigational Drug | 3 |  |
| Unknown [N11] | 3 |  |
| Medicated Dressings | 2 |  |
| Unknown [D12] | 2 |  |
| Unknown [J11] | 2 |  |
| Unknown [V999] | 2 |  |
| Appetite Stimulants | 1 |  |
