# Supplementary material for: The potential antidepressant effect of antidiabetic agents: New insights from a pharmacovigilance study based on data from the reporting system databases FAERS and VigiBase
Source: Front Pharmacol. 2023 Feb 17;14:1128387. doi: 10.3389/fphar.2023.1128387 (PMC9981969; doi:10.3389/fphar.2023.1128387)
Supplement: Supplementary file 2 [file Table2.DOCX]

**STable 2.** Number of medications reported as suspect (either primary or secondary suspect) drugs, grouping by ATC Level 2, for *cases* (a) and *non-cases* (b), in the VigiBase.

| **a) *CASES*** | | |
| --- | --- | --- |
| **ATC Level 2** | **Drug references** | |
| Psychoanaleptics | 135471 | |
| Psycholeptics | 43674 | |
| Analgesics | 17534 | |
| Antiepileptics | 12419 | |
| Drugs For Acid Related Disorders | 4158 | |
| Agents Acting On The Renin-Angiotensin System | 3950 | |
| Lipid Modifying Agents | 3882 | |
| Antiinflammatory And Antirheumatic Products | 3837 | |
| Beta Blocking Agents | 3529 | |
| Cardiac Therapy | 3487 | |
| Unclassified | 3425 | |
| Muscle Relaxants | 3033 | |
| Drugs Used In Diabetes | 2929 | |
| Thyroid Therapy | 2895 | |
| Antithrombotic Agents | 2712 | |
| Calcium Channel Blockers | 2548 | |
| Antihistamines For Systemic Use | 2477 | |
| Vitamins | 2395 | |
| Drugs For Obstructive Airway Diseases | 2364 | |
| Other Nervous System Drugs | 2354 | |
| Sex Hormones And Modulators Of The Genital System | 1977 | |
| Diuretics | 1870 | |
| Immunosuppressants | 1669 | |
| Ophthalmologicals | 1499 | |
| Other Dermatological Preparations | 1428 | |
| Antineoplastic Agents | 1314 | |
| Cough And Cold Preparations | 1281 | |
| Antibacterials For Systemic Use | 1274 | |
| Anti-Parkinson Drugs | 1258 | |
| Urologicals | 1252 | |
| Topical Products For Joint And Muscular Pain | 1078 | |
| Corticosteroids For Systemic Use | 994 | |
| Unspecified Herbal And Traditional Medicine | 930 | |
| Nasal Preparations | 886 | |
| Mineral Supplements | 841 | |
| Antiemetics And Antinauseants | 829 | |
| Drugs For Constipation | 723 | |
| Drugs For Functional Gastrointestinal Disorders | 719 | |
| Antianemic Preparations | 680 | |
| Antivirals For Systemic Use | 627 | |
| Antipruritics, Incl. Antihistamines, Anesthetics, Etc. | 626 | |
| Stomatological Preparations | 472 | |
| Homeopathic Preparation | 444 | |
| Antidiarrheals, Intestinal Antiinflammatory/Antiinfective Agents | 426 | |
| Anesthetics | 411 | |
| All Other Therapeutic Products | 374 | |
| Immunostimulants | 365 | |
| Blood Substitutes And Perfusion Solutions | 311 | |
| Drugs For Treatment Of Bone Diseases | 307 | |
| Antihypertensives | 269 | |
| Endocrine Therapy | 249 | |
| Antiobesity Preparations, Excl. Diet Products | 198 | |
| Antigout Preparations | 192 | |
| Anti-Acne Preparations | 158 | |
| Antifungals For Dermatological Use | 135 | |
| Vaccines | 97 | |
| Antiseptics And Disinfectants | 91 | |
| Other Alimentary Tract And Metabolism Products | 90 | |
| Other Gynecologicals | 88 | |
| Pituitary And Hypothalamic Hormones And Analogues | 81 | |
| Calcium Homeostasis | 74 | |
| Vasoprotectives | 71 | |
| Digestives, Incl. Enzymes | 71 | |
| Diagnostic Agents | 64 | |
| Corticosteroids, Dermatological Preparations | 63 | |
| Antiprotozoals | 56 | |
| Unknown [C999] | 53 | |
| Antibiotics And Chemotherapeutics For Dermatological Use | 50 | |
| Bile And Liver Therapy | 46 | |
| Antimycotics For Systemic Use | 43 | |
| General Nutrients | 39 | |
| Peripheral Vasodilators | 38 | |
| Investigational Drug | 33 | |
| Unknown [V999] | 32 | |
| Antimycobacterials | 32 | |
| Immune Sera And Immunoglobulins | 30 | |
| Antipsoriatics | 27 | |
| Anthelmintics | 27 | |
| Antihemorrhagics | 26 | |
| Anabolic Agents For Systemic Use | 24 | |
| All Other Non-Therapeutic Products | 24 | |
| Emollients And Protectives | 22 | |
| Unknown [N999] | 20 | |
| Other Respiratory System Products | 19 | |
| Ectoparasiticides, Incl. Scabicides, Insecticides And Repellents | 18 | |
| Preparations For Treatment Of Wounds And Ulcers | 17 | |
| Other Hematological Agents | 15 | |
| Pancreatic Hormones | 14 | |
| Gynecological Antiinfectives And Antiseptics | 12 | |
| Tonics | 11 | |
| Throat Preparations | 8 | |
| Unknown [L999] | 7 | |
| Otologicals | 6 | |
| Other Drugs For Disorders Of The Musculo-Skeletal System | 6 | |
| Contrast Media | 4 | |
| Allergens | 4 | |
| Unknown [R999] | 3 | |
| Diagnostic Radiopharmaceuticals | 3 | |
| Unknown [H999] | 2 | |
| Unknown [J999] | 1 | |
| Unknown [A999] | 1 | |
| Therapeutic Radiopharmaceuticals | 1 | |
| Ophthalmological And Otological Preparations | 1 | |
| Appetite Stimulants | 1 | |
| ***b) NON-CASES*** | | |
| **ATC Level 2** | | **Drug references** |
| Psychoanaleptics | | 721133 |
| Psycholeptics | | 190170 |
| Analgesics | | 72369 |
| Antiepileptics | | 44415 |
| Drugs For Acid Related Disorders | | 37088 |
| Antithrombotic Agents | | 29065 |
| Agents Acting On The Renin-Angiotensin System | | 27449 |
| Lipid Modifying Agents | | 25875 |
| Unclassified | | 23778 |
| Beta Blocking Agents | | 22823 |
| Diuretics | | 21866 |
| Sex Hormones And Modulators Of The Genital System | | 20508 |
| Drugs For Obstructive Airway Diseases | | 20052 |
| Antibacterials For Systemic Use | | 19732 |
| Drugs Used In Diabetes | | 19094 |
| Thyroid Therapy | | 19063 |
| Antiinflammatory And Antirheumatic Products | | 18596 |
| Cardiac Therapy | | 15364 |
| Vitamins | | 14442 |
| Calcium Channel Blockers | | 14425 |
| Antihistamines For Systemic Use | | 12707 |
| Ophthalmologicals | | 12529 |
| Anti-Parkinson Drugs | | 10232 |
| Other Nervous System Drugs | | 9316 |
| Muscle Relaxants | | 9283 |
| Drugs For Constipation | | 9062 |
| Corticosteroids For Systemic Use | | 8281 |
| Drugs For Functional Gastrointestinal Disorders | | 8194 |
| Urologicals | | 7860 |
| Nasal Preparations | | 7804 |
| Mineral Supplements | | 7304 |
| Antineoplastic Agents | | 7283 |
| Immunosuppressants | | 7140 |
| Other Dermatological Preparations | | 7118 |
| Topical Products For Joint And Muscular Pain | | 7087 |
| Cough And Cold Preparations | | 6753 |
| Unspecified Herbal And Traditional Medicine | | 6515 |
| Antianemic Preparations | | 5991 |
| Antiemetics And Antinauseants | | 4554 |
| Antivirals For Systemic Use | | 4259 |
| Antihypertensives | | 2960 |
| Antidiarrheals, Intestinal Antiinflammatory/Antiinfective Agents | | 2886 |
| Drugs For Treatment Of Bone Diseases | | 2860 |
| Homeopathic Preparation | | 2758 |
| Stomatological Preparations | | 2757 |
| Antiobesity Preparations, Excl. Diet Products | | 2630 |
| Antigout Preparations | | 2627 |
| Blood Substitutes And Perfusion Solutions | | 2274 |
| Endocrine Therapy | | 2233 |
| Antipruritics, Incl. Antihistamines, Anesthetics, Etc. | | 2090 |
| Anesthetics | | 1896 |
| Immunostimulants | | 1675 |
| Antifungals For Dermatological Use | | 1395 |
| All Other Therapeutic Products | | 1152 |
| Anti-Acne Preparations | | 1054 |
| Peripheral Vasodilators | | 1001 |
| Vaccines | | 935 |
| Vasoprotectives | | 858 |
| Antimycotics For Systemic Use | | 755 |
| Other Alimentary Tract And Metabolism Products | | 721 |
| Calcium Homeostasis | | 661 |
| Corticosteroids, Dermatological Preparations | | 652 |
| Other Gynecologicals | | 587 |
| Antiprotozoals | | 581 |
| Bile And Liver Therapy | | 512 |
| Digestives, Incl. Enzymes | | 484 |
| Antibiotics And Chemotherapeutics For Dermatological Use | | 481 |
| Antimycobacterials | | 477 |
| Pituitary And Hypothalamic Hormones And Analogues | | 426 |
| Emollients And Protectives | | 380 |
| Antihemorrhagics | | 357 |
| General Nutrients | | 354 |
| Gynecological Antiinfectives And Antiseptics | | 296 |
| Investigational Drug | | 283 |
| Antipsoriatics | | 253 |
| Immune Sera And Immunoglobulins | | 232 |
| Unknown [C999] | | 208 |
| Antiseptics And Disinfectants | | 161 |
| Anabolic Agents For Systemic Use | | 135 |
| Contrast Media | | 119 |
| Unknown [V999] | | 112 |
| Otologicals | | 102 |
| Other Drugs For Disorders Of The Musculo-Skeletal System | | 92 |
| All Other Non-Therapeutic Products | | 80 |
| Tonics | | 80 |
| Anthelmintics | | 78 |
| Preparations For Treatment Of Wounds And Ulcers | | 73 |
| Diagnostic Agents | | 70 |
| Throat Preparations | | 69 |
| Ectoparasiticides, Incl. Scabicides, Insecticides And Repellents | | 67 |
| Other Respiratory System Products | | 58 |
| Unknown [A999] | | 48 |
| Other Hematological Agents | | 45 |
| Appetite Stimulants | | 37 |
| Pancreatic Hormones | | 32 |
| Unknown [N999] | | 32 |
| Unknown [L999] | | 31 |
| Allergens | | 28 |
| Diagnostic Radiopharmaceuticals | | 20 |
| Ophthalmological And Otological Preparations | | 16 |
| Therapeutic Radiopharmaceuticals | | 14 |
| Medicated Dressings | | 7 |
| Unknown [J999] | | 5 |
| Unknown [R999] | | 4 |
| Unknown [G999] | | 3 |
| Unknown [D999] | | 2 |
| Unknown [H999] | | 2 |
| Unknown [M999] | | 2 |
