# Supplementary material for: The potential antidepressant effect of antidiabetic agents: New insights from a pharmacovigilance study based on data from the reporting system databases FAERS and VigiBase
Source: Front Pharmacol. 2023 Feb 17;14:1128387. doi: 10.3389/fphar.2023.1128387 (PMC9981969; doi:10.3389/fphar.2023.1128387)
Supplement: Supplementary file 3 [file Table3.DOCX]

**STable 3.** Disproportionality scores for cases and non-cases exposed to antidiabetic drug classes, in the FAERS.

| **Active substance or ATC code** | **ATC code** | **SMQ** | **N** | **ROR** | **ROR05** | **ROR95** | **ERAM** | **ER05** | **ER95** | **EBGM** | **EB05** | **EB95** | **PRR** | **P_VALUE** |
| --- | --- | --- | --- | --- | --- | --- | --- | --- | --- | --- | --- | --- | --- | --- |
| A10BA Biguanides | Biguanides | Cases - Therapy failure | 1727 | 1,085 | 1,038 | 1,135 | 0,856 | 0,823 | 0,890 | 0,919 | 0,884 | 0,956 | 1,068 | 0,003 |
| A10BA Biguanides | Biguanides | Non-cases - Therapy failure | 7017 | 0,922 | 0,881 | 0,964 | 0,980 | 0,961 | 0,999 | 1,022 | 1,002 | 1,042 | 0,984 | 0,003 |
| A10BB Sulfonylureas | Sulfonylureas | Cases - Therapy failure | 696 | 0,831 | 0,776 | 0,890 | 0,858 | 0,805 | 0,912 | 0,935 | 0,878 | 0,995 | 0,858 | 0,000 |
| A10BB Sulfonylureas | Sulfonylureas | Non-cases - Therapy failure | 3682 | 1,203 | 1,124 | 1,288 | 0,985 | 0,960 | 1,011 | 1,013 | 0,986 | 1,041 | 1,032 | 0,000 |
| A10BG Thiazolidinediones | Thiazolidinediones | Cases - Therapy failure | 187 | 0,925 | 0,810 | 1,056 | 0,818 | 0,723 | 0,918 | 0,919 | 0,815 | 1,034 | 0,938 | 0,353 |
| A10BG Thiazolidinediones | Thiazolidinediones | Non-cases - Therapy failure | 890 | 1,081 | 0,947 | 1,234 | 0,993 | 0,945 | 1,041 | 1,020 | 0,965 | 1,077 | 1,014 | 0,353 |
| A10BH DPP4-inhibitors | Dipeptidyl Peptidase 4 (Dpp-4) Inhibitors | Cases - Therapy failure | 214 | 0,761 | 0,674 | 0,860 | 0,676 | 0,602 | 0,753 | 0,687 | 0,614 | 0,767 | 0,796 | 0,000 |
| A10BH DPP4-inhibitors | Dipeptidyl Peptidase 4 (Dpp-4) Inhibitors | Non-cases - Therapy failure | 1237 | 1,314 | 1,163 | 1,484 | 1,034 | 0,991 | 1,078 | 1,087 | 1,037 | 1,139 | 1,046 | 0,000 |
| A10BJ GLP-1 analogues | Glucagon-Like Peptide-1 (Glp-1) Analogues | Cases - Therapy failure | 82 | 0,546 | 0,450 | 0,662 | 0,480 | 0,398 | 0,569 | 0,488 | 0,407 | 0,582 | 0,596 | 0,000 |
| A10BJ GLP-1 analogues | Glucagon-Like Peptide-1 (Glp-1) Analogues | Non-cases - Therapy failure | 661 | 1,832 | 1,511 | 2,221 | 1,065 | 1,008 | 1,123 | 1,156 | 1,084 | 1,231 | 1,092 | 0,000 |
| A10BK Sodium-glucose co-transporter 2 (SGLT2) inhibitors | Sodium-Glucose Co-Transporter 2 (Sglt2) Inhibitors | Cases - Therapy failure | 52 | 0,901 | 0,702 | 1,158 | 0,715 | 0,564 | 0,881 | 0,716 | 0,571 | 0,890 | 0,918 | 0,543 |
| A10BKSodium-glucose co-transporter 2 (SGLT2) inhibitors | Sodium-Glucose Co-Transporter 2 (Sglt2) Inhibitors | Non-cases - Therapy failure | 254 | 1,109 | 0,864 | 1,425 | 1,008 | 0,937 | 1,082 | 1,095 | 0,987 | 1,212 | 1,019 | 0,543 |
| Canagliflozin | Sodium-Glucose Co-Transporter 2 (Sglt2) Inhibitors | Cases - Therapy failure | 19 | 1,268 | 0,826 | 1,945 | 0,894 | 0,605 | 1,229 | 0,952 | 0,662 | 1,335 | 1,208 | 0,439 |
| Canagliflozin | Sodium-Glucose Co-Transporter 2 (Sglt2) Inhibitors | Non-cases - Therapy failure | 66 | 0,789 | 0,514 | 1,211 | 0,992 | 0,905 | 1,083 | 1,025 | 0,838 | 1,245 | 0,953 | 0,439 |
| Chlorpropamide | Sulfonylureas | Cases - Therapy failure | 5 | 0,253 | 0,119 | 0,539 | 0,767 | 0,382 | 1,260 | 0,819 | 0,441 | 1,416 | 0,294 | 0,002 |
| Chlorpropamide | Sulfonylureas | Non-cases - Therapy failure | 87 | 3,952 | 1,855 | 8,422 | 1,011 | 0,925 | 1,101 | 1,027 | 0,861 | 1,217 | 1,160 | 0,002 |
| Dapagliflozin | Sodium-Glucose Co-Transporter 2 (Sglt2) Inhibitors | Cases - Therapy failure | 9 | 0,543 | 0,304 | 0,971 | 0,573 | 0,331 | 0,870 | 0,565 | 0,343 | 0,888 | 0,593 | 0,106 |
| Dapagliflozin | Sodium-Glucose Co-Transporter 2 (Sglt2) Inhibitors | Non-cases - Therapy failure | 73 | 1,842 | 1,030 | 3,294 | 1,017 | 0,928 | 1,109 | 1,148 | 0,947 | 1,381 | 1,092 | 0,106 |
| Dulaglutide | Glucagon-Like Peptide-1 (Glp-1) Analogues | Cases - Therapy failure | 10 | 0,373 | 0,217 | 0,641 | 0,382 | 0,226 | 0,571 | 0,375 | 0,232 | 0,580 | 0,422 | 0,003 |
| Dulaglutide | Glucagon-Like Peptide-1 (Glp-1) Analogues | Non-cases - Therapy failure | 118 | 2,680 | 1,559 | 4,608 | 1,021 | 0,937 | 1,108 | 1,223 | 1,051 | 1,417 | 1,131 | 0,003 |
| Empagliflozin | Sodium-Glucose Co-Transporter 2 (Sglt2) Inhibitors | Cases - Therapy failure | 18 | 0,911 | 0,595 | 1,395 | 0,723 | 0,485 | 1,001 | 0,721 | 0,496 | 1,018 | 0,926 | 0,815 |
| Empagliflozin | Sodium-Glucose Co-Transporter 2 (Sglt2) Inhibitors | Non-cases - Therapy failure | 87 | 1,098 | 0,717 | 1,681 | 0,998 | 0,913 | 1,087 | 1,107 | 0,928 | 1,313 | 1,017 | 0,815 |
| Exenatide | Glucagon-Like Peptide-1 (Glp-1) Analogues | Cases - Therapy failure | 30 | 0,791 | 0,571 | 1,096 | 0,649 | 0,475 | 0,845 | 0,711 | 0,529 | 0,938 | 0,823 | 0,274 |
| Exenatide | Glucagon-Like Peptide-1 (Glp-1) Analogues | Non-cases - Therapy failure | 167 | 1,264 | 0,912 | 1,752 | 0,990 | 0,913 | 1,069 | 1,090 | 0,959 | 1,234 | 1,040 | 0,274 |
| Gliclazide | Sulfonylureas | Cases - Therapy failure | 99 | 0,527 | 0,443 | 0,628 | 0,552 | 0,465 | 0,645 | 0,556 | 0,471 | 0,653 | 0,578 | 0,000 |
| Gliclazide | Sulfonylureas | Non-cases - Therapy failure | 826 | 1,897 | 1,592 | 2,259 | 1,035 | 0,984 | 1,087 | 1,110 | 1,048 | 1,174 | 1,096 | 0,000 |
| Glimepiride | Sulfonylureas | Cases - Therapy failure | 149 | 0,937 | 0,808 | 1,087 | 0,853 | 0,742 | 0,970 | 0,918 | 0,802 | 1,047 | 0,948 | 0,500 |
| Glimepiride | Sulfonylureas | Non-cases - Therapy failure | 700 | 1,067 | 0,920 | 1,238 | 0,995 | 0,943 | 1,048 | 1,021 | 0,959 | 1,085 | 1,012 | 0,500 |
| Glipizide | Sulfonylureas | Cases - Therapy failure | 206 | 1,123 | 0,987 | 1,277 | 1,043 | 0,927 | 1,164 | 1,186 | 1,057 | 1,327 | 1,098 | 0,149 |
| Glipizide | Sulfonylureas | Non-cases - Therapy failure | 808 | 0,891 | 0,783 | 1,013 | 0,962 | 0,914 | 1,011 | 0,962 | 0,908 | 1,019 | 0,978 | 0,149 |
| Gliquidone | Sulfonylureas | Cases - Therapy failure | 1 | 0,440 | 0,078 | 2,472 | 0,806 | 0,275 | 1,563 | 0,864 | 0,352 | 1,844 | 0,491 | 0,677 |
| Gliquidone | Sulfonylureas | Non-cases - Therapy failure | 10 | 2,271 | 0,405 | 12,750 | 1,002 | 0,905 | 1,102 | 1,104 | 0,684 | 1,706 | 1,116 | 0,677 |
| Linagliptin | Dipeptidyl Peptidase 4 (Dpp-4) Inhibitors | Cases - Therapy failure | 19 | 0,669 | 0,446 | 1,003 | 0,654 | 0,443 | 0,900 | 0,651 | 0,452 | 0,913 | 0,713 | 0,125 |
| Linagliptin | Dipeptidyl Peptidase 4 (Dpp-4) Inhibitors | Non-cases - Therapy failure | 125 | 1,494 | 0,997 | 2,241 | 0,999 | 0,918 | 1,083 | 1,107 | 0,955 | 1,277 | 1,065 | 0,125 |
| Liraglutide | Glucagon-Like Peptide-1 (Glp-1) Analogues | Cases - Therapy failure | 39 | 0,580 | 0,438 | 0,768 | 0,529 | 0,403 | 0,670 | 0,534 | 0,411 | 0,683 | 0,629 | 0,002 |
| Liraglutide | Glucagon-Like Peptide-1 (Glp-1) Analogues | Non-cases - Therapy failure | 296 | 1,724 | 1,303 | 2,282 | 1,010 | 0,941 | 1,081 | 1,142 | 1,038 | 1,255 | 1,084 | 0,002 |
| Lixisenatide | Glucagon-Like Peptide-1 (Glp-1) Analogues | Cases - Therapy failure | 1 | 0,629 | 0,108 | 3,651 | 0,883 | 0,302 | 1,712 | 0,871 | 0,355 | 1,858 | 0,675 | 0,986 |
| Lixisenatide | Glucagon-Like Peptide-1 (Glp-1) Analogues | Non-cases - Therapy failure | 7 | 1,590 | 0,274 | 9,227 | 0,999 | 0,903 | 1,100 | 1,136 | 0,656 | 1,859 | 1,074 | 0,986 |
| Metformin | Biguanides | Cases - Therapy failure | 1645 | 1,107 | 1,057 | 1,159 | 0,866 | 0,831 | 0,901 | 0,933 | 0,896 | 0,972 | 1,085 | 0,000 |
| Metformin | Biguanides | Non-cases - Therapy failure | 6554 | 0,903 | 0,863 | 0,946 | 0,999 | 0,979 | 1,019 | 1,018 | 0,998 | 1,039 | 0,981 | 0,000 |
| Pioglitazone | Thiazolidinediones | Cases - Therapy failure | 116 | 1,030 | 0,869 | 1,220 | 0,821 | 0,701 | 0,948 | 0,934 | 0,802 | 1,084 | 1,024 | 0,816 |
| Pioglitazone | Thiazolidinediones | Non-cases - Therapy failure | 496 | 0,971 | 0,819 | 1,151 | 0,989 | 0,931 | 1,048 | 1,018 | 0,945 | 1,095 | 0,995 | 0,816 |
| Rosiglitazone | Thiazolidinediones | Cases - Therapy failure | 58 | 0,909 | 0,717 | 1,152 | 0,815 | 0,651 | 0,993 | 0,923 | 0,744 | 1,134 | 0,924 | 0,553 |
| Rosiglitazone | Thiazolidinediones | Non-cases - Therapy failure | 281 | 1,100 | 0,868 | 1,395 | 0,997 | 0,928 | 1,068 | 1,020 | 0,925 | 1,124 | 1,017 | 0,553 |
| Saxagliptin | Dipeptidyl Peptidase 4 (Dpp-4) Inhibitors | Cases - Therapy failure | 6 | 0,372 | 0,185 | 0,749 | 0,488 | 0,254 | 0,782 | 0,485 | 0,271 | 0,815 | 0,421 | 0,023 |
| Saxagliptin | Dipeptidyl Peptidase 4 (Dpp-4) Inhibitors | Non-cases - Therapy failure | 71 | 2,688 | 1,335 | 5,409 | 1,015 | 0,926 | 1,107 | 1,157 | 0,952 | 1,395 | 1,132 | 0,023 |
| Semaglutide | Glucagon-Like Peptide-1 (Glp-1) Analogues | Cases - Therapy failure | 4 | 0,267 | 0,114 | 0,622 | 0,330 | 0,155 | 0,558 | 0,349 | 0,178 | 0,626 | 0,309 | 0,009 |
| Semaglutide | Glucagon-Like Peptide-1 (Glp-1) Analogues | Non-cases - Therapy failure | 66 | 3,748 | 1,606 | 8,743 | 1,012 | 0,923 | 1,105 | 1,242 | 1,014 | 1,507 | 1,157 | 0,009 |
| Sitagliptin | Dipeptidyl Peptidase 4 (Dpp-4) Inhibitors | Cases - Therapy failure | 139 | 0,968 | 0,830 | 1,130 | 0,809 | 0,701 | 0,924 | 0,840 | 0,730 | 0,962 | 0,974 | 0,767 |
| Sitagliptin | Dipeptidyl Peptidase 4 (Dpp-4) Inhibitors | Non-cases - Therapy failure | 632 | 1,033 | 0,885 | 1,205 | 0,979 | 0,926 | 1,033 | 1,045 | 0,979 | 1,115 | 1,006 | 0,767 |
| Tolazamide | Sulfonylureas | Cases - Therapy failure | 1 | 0,315 | 0,057 | 1,726 | 0,877 | 0,300 | 1,701 | 0,986 | 0,403 | 2,103 | 0,360 | 0,396 |
| Tolazamide | Sulfonylureas | Non-cases - Therapy failure | 14 | 3,180 | 0,579 | 17,453 | 1,002 | 0,906 | 1,101 | 1,041 | 0,687 | 1,526 | 1,145 | 0,396 |
| Troglitazone | Thiazolidinediones | Cases - Therapy failure | 6 | 0,318 | 0,159 | 0,638 | 0,717 | 0,374 | 1,151 | 0,808 | 0,452 | 1,357 | 0,364 | 0,006 |
| Troglitazone | Thiazolidinediones | Non-cases - Therapy failure | 83 | 3,142 | 1,567 | 6,298 | 1,003 | 0,917 | 1,092 | 1,033 | 0,862 | 1,229 | 1,144 | 0,006 |
| Vildagliptin | Dipeptidyl Peptidase 4 (Dpp-4) Inhibitors | Cases - Therapy failure | 6 | 0,304 | 0,152 | 0,608 | 0,458 | 0,239 | 0,734 | 0,421 | 0,235 | 0,707 | 0,349 | 0,004 |
| Vildagliptin | Dipeptidyl Peptidase 4 (Dpp-4) Inhibitors | Non-cases - Therapy failure | 87 | 3,293 | 1,645 | 6,595 | 1,017 | 0,930 | 1,107 | 1,166 | 0,977 | 1,382 | 1,148 | 0,004 |
