# Supplementary material for: The potential antidepressant effect of antidiabetic agents: New insights from a pharmacovigilance study based on data from the reporting system databases FAERS and VigiBase
Source: Front Pharmacol. 2023 Feb 17;14:1128387. doi: 10.3389/fphar.2023.1128387 (PMC9981969; doi:10.3389/fphar.2023.1128387)
Supplement: Supplementary file 4 [file Table4.docx]

**STable 4.** Disproportionality scores for cases and non-cases exposed to antidiabetic drug classes, in the VigiBase.

| **ATC code or active substance** | **ATC4** | **SMQ** | **N** | **ROR** | **ROR05** | **ROR95** | **PRR** | **P_VALUE** | **ERAM** | **ER05** | **ER95** | **EBGM** | **EB05** | **EB95** |
| --- | --- | --- | --- | --- | --- | --- | --- | --- | --- | --- | --- | --- | --- | --- |
| A10BA Biguanides | Biguanides | Cases - Therapy failure | 1282 | 1,411 | 1,341 | 1,485 | 1,339 | 0,000 | 0,822 | 0,784 | 0,860 | 1,163 | 1,110 | 1,217 |
| A10BA Biguanides | Biguanides | Non-cases - Therapy failure | 6014 | 0,709 | 0,673 | 0,746 | 0,949 | 0,000 | 0,954 | 0,934 | 0,974 | 0,971 | 0,951 | 0,992 |
| A10BB Sulfonylureas | Sulfonylureas | Cases - Therapy failure | 442 | 0,830 | 0,764 | 0,902 | 0,849 | 0,000 | 0,838 | 0,773 | 0,904 | 1,010 | 0,934 | 1,092 |
| A10BB Sulfonylureas | Sulfonylureas | Non-cases - Therapy failure | 3506 | 1,205 | 1,108 | 1,309 | 1,023 | 0,000 | 0,988 | 0,962 | 1,014 | 0,999 | 0,972 | 1,027 |
| A10BG Thiazolidinediones | Thiazolidinediones | Cases - Therapy failure | 144 | 1,376 | 1,184 | 1,600 | 1,311 | 0,001 | 0,926 | 0,804 | 1,055 | 1,176 | 1,025 | 1,344 |
| A10BG Thiazolidinediones | Thiazolidinediones | Non-cases - Therapy failure | 690 | 0,727 | 0,625 | 0,845 | 0,953 | 0,001 | 0,968 | 0,921 | 1,016 | 0,970 | 0,911 | 1,032 |
| A10BH DPP4-inhibitors | DPP4-inhibitors | Cases - Therapy failure | 115 | 0,900 | 0,764 | 1,060 | 0,912 | 0,312 | 0,765 | 0,653 | 0,884 | 0,840 | 0,720 | 0,974 |
| A10BH DPP4-inhibitors | DPP4-inhibitors | Non-cases - Therapy failure | 842 | 1,111 | 0,943 | 1,308 | 1,013 | 0,312 | 1,000 | 0,954 | 1,046 | 1,029 | 0,972 | 1,088 |
| A10BJ GLP-1 analogues | GLP-1 analogues | Cases - Therapy failure | 48 | 0,717 | 0,559 | 0,921 | 0,745 | 0,033 | 0,515 | 0,403 | 0,639 | 0,586 | 0,464 | 0,733 |
| A10BJ GLP-1 analogues | GLP-1 analogues | Non-cases - Therapy failure | 441 | 1,394 | 1,086 | 1,790 | 1,039 | 0,033 | 1,028 | 0,971 | 1,086 | 1,092 | 1,009 | 1,179 |
| A10BK Sodium-glucose co-transporter 2 (SGLT2) inhibitors | Sodium-Glucose Co-Transporter 2 (Sglt2) Inhibitors | Cases - Therapy failure | 34 | 1,401 | 1,027 | 1,911 | 1,331 | 0,092 | 0,918 | 0,685 | 1,179 | 0,994 | 0,755 | 1,289 |
| A10BKSodium-glucose co-transporter 2 (SGLT2) inhibitors | Sodium-Glucose Co-Transporter 2 (Sglt2) Inhibitors | Non-cases - Therapy failure | 160 | 0,714 | 0,523 | 0,974 | 0,950 | 0,092 | 0,990 | 0,924 | 1,058 | 1,006 | 0,883 | 1,142 |
| Canagliflozin | Sodium-Glucose Co-Transporter 2 (Sglt2) Inhibitors | Cases - Therapy failure | 14 | 2,797 | 1,655 | 4,726 | 2,262 | 0,002 | 1,169 | 0,746 | 1,670 | 1,466 | 0,977 | 2,131 |
| Canagliflozin | Sodium-Glucose Co-Transporter 2 (Sglt2) Inhibitors | Non-cases - Therapy failure | 33 | 0,358 | 0,212 | 0,604 | 0,809 | 0,002 | 0,986 | 0,912 | 1,062 | 0,884 | 0,669 | 1,150 |
| Chlorpropamide | Sulfonylureas | Cases - Therapy failure | 1 | 0,092 | 0,017 | 0,480 | 0,104 | 0,005 | 0,715 | 0,247 | 1,380 | 0,888 | 0,393 | 1,784 |
| Chlorpropamide | Sulfonylureas | Non-cases - Therapy failure | 72 | 10,924 | 2,085 | 57,248 | 1,136 | 0,005 | 1,005 | 0,933 | 1,080 | 1,019 | 0,840 | 1,226 |
| Dapagliflozin | Sodium-Glucose Co-Transporter 2 (Sglt2) Inhibitors | Cases - Therapy failure | 6 | 0,791 | 0,389 | 1,610 | 0,813 | 0,729 | 0,659 | 0,345 | 1,055 | 0,714 | 0,410 | 1,175 |
| Dapagliflozin | Sodium-Glucose Co-Transporter 2 (Sglt2) Inhibitors | Non-cases - Therapy failure | 50 | 1,264 | 0,621 | 2,573 | 1,028 | 0,729 | 1,005 | 0,931 | 1,081 | 1,096 | 0,871 | 1,364 |
| Dulaglutide | Glucagon-Like Peptide-1 (Glp-1) Analogues | Cases - Therapy failure | 8 | 0,722 | 0,391 | 1,333 | 0,750 | 0,476 | 0,629 | 0,354 | 0,969 | 0,668 | 0,403 | 1,053 |
| Dulaglutide | Glucagon-Like Peptide-1 (Glp-1) Analogues | Non-cases - Therapy failure | 73 | 1,384 | 0,750 | 2,555 | 1,038 | 0,476 | 1,008 | 0,935 | 1,082 | 1,095 | 0,904 | 1,316 |
| Empagliflozin | Sodium-Glucose Co-Transporter 2 (Sglt2) Inhibitors | Cases - Therapy failure | 13 | 1,279 | 0,777 | 2,106 | 1,234 | 0,517 | 0,945 | 0,593 | 1,363 | 0,966 | 0,636 | 1,419 |
| Empagliflozin | Sodium-Glucose Co-Transporter 2 (Sglt2) Inhibitors | Non-cases - Therapy failure | 67 | 0,782 | 0,475 | 1,287 | 0,965 | 0,517 | 0,996 | 0,924 | 1,071 | 1,020 | 0,836 | 1,236 |
| Exenatide | Glucagon-Like Peptide-1 (Glp-1) Analogues | Cases - Therapy failure | 24 | 1,106 | 0,770 | 1,590 | 1,091 | 0,731 | 0,722 | 0,510 | 0,964 | 0,864 | 0,626 | 1,169 |
| Exenatide | Glucagon-Like Peptide-1 (Glp-1) Analogues | Non-cases - Therapy failure | 143 | 0,904 | 0,629 | 1,299 | 0,986 | 0,731 | 1,002 | 0,934 | 1,072 | 1,037 | 0,903 | 1,185 |
| Gliclazide | Sulfonylureas | Cases - Therapy failure | 40 | 0,310 | 0,238 | 0,405 | 0,341 | 0,000 | 0,438 | 0,334 | 0,553 | 0,439 | 0,340 | 0,559 |
| Gliclazide | Sulfonylureas | Non-cases - Therapy failure | 849 | 3,223 | 2,470 | 4,207 | 1,100 | 0,000 | 1,032 | 0,985 | 1,079 | 1,071 | 1,012 | 1,133 |
| Glimepiride | Sulfonylureas | Cases - Therapy failure | 113 | 0,984 | 0,833 | 1,162 | 0,986 | 0,912 | 0,851 | 0,726 | 0,985 | 0,969 | 0,830 | 1,126 |
| Glimepiride | Sulfonylureas | Non-cases - Therapy failure | 757 | 1,016 | 0,861 | 1,200 | 1,002 | 0,912 | 0,991 | 0,945 | 1,039 | 1,006 | 0,947 | 1,067 |
| Glipizide | Sulfonylureas | Cases - Therapy failure | 168 | 1,502 | 1,304 | 1,728 | 1,409 | 0,000 | 1,087 | 0,954 | 1,228 | 1,431 | 1,260 | 1,619 |
| Glipizide | Sulfonylureas | Non-cases - Therapy failure | 738 | 0,666 | 0,579 | 0,767 | 0,938 | 0,000 | 0,950 | 0,905 | 0,996 | 0,936 | 0,881 | 0,994 |
| Gliquidone | Sulfonylureas | Cases - Therapy failure | 1 | 0,942 | 0,162 | 5,466 | 0,949 | 0,641 | 1,026 | 0,355 | 1,980 | 1,185 | 0,527 | 2,375 |
| Gliquidone | Sulfonylureas | Non-cases - Therapy failure | 7 | 1,062 | 0,183 | 6,164 | 1,008 | 0,641 | 0,999 | 0,922 | 1,078 | 1,007 | 0,595 | 1,619 |
| Linagliptin | Dipeptidyl Peptidase 4 (Dpp-4) Inhibitors | Cases - Therapy failure | 11 | 0,642 | 0,382 | 1,079 | 0,673 | 0,199 | 0,645 | 0,390 | 0,951 | 0,720 | 0,460 | 1,084 |
| Linagliptin | Dipeptidyl Peptidase 4 (Dpp-4) Inhibitors | Non-cases - Therapy failure | 113 | 1,559 | 0,927 | 2,621 | 1,050 | 0,199 | 1,005 | 0,935 | 1,076 | 1,059 | 0,908 | 1,230 |
| Liraglutide | Glucagon-Like Peptide-1 (Glp-1) Analogues | Cases - Therapy failure | 17 | 0,519 | 0,343 | 0,785 | 0,554 | 0,011 | 0,414 | 0,275 | 0,577 | 0,472 | 0,324 | 0,668 |
| Liraglutide | Glucagon-Like Peptide-1 (Glp-1) Analogues | Non-cases - Therapy failure | 216 | 1,928 | 1,274 | 2,918 | 1,068 | 0,011 | 1,027 | 0,962 | 1,094 | 1,122 | 1,003 | 1,252 |
| Lixisenatide | Glucagon-Like Peptide-1 (Glp-1) Analogues | Cases - Therapy failure | 1 | 2,197 | 0,329 | 14,683 | 1,898 | 0,968 | 1,089 | 0,377 | 2,102 | 1,160 | 0,516 | 2,326 |
| Lixisenatide | Glucagon-Like Peptide-1 (Glp-1) Analogues | Non-cases - Therapy failure | 3 | 0,455 | 0,068 | 3,041 | 0,864 | 0,968 | 0,999 | 0,922 | 1,079 | 1,025 | 0,523 | 1,849 |
| Metformin | Biguanides | Cases - Therapy failure | 1280 | 1,420 | 1,349 | 1,494 | 1,345 | 0,000 | 1,200 | 1,145 | 1,255 | 1,166 | 1,113 | 1,220 |
| Metformin | Biguanides | Non-cases - Therapy failure | 5970 | 0,704 | 0,669 | 0,741 | 0,948 | 0,000 | 0,953 | 0,934 | 0,973 | 0,970 | 0,950 | 0,991 |
| Pioglitazone | Thiazolidinediones | Cases - Therapy failure | 87 | 1,382 | 1,138 | 1,678 | 1,316 | 0,007 | 0,883 | 0,736 | 1,042 | 1,121 | 0,940 | 1,328 |
| Pioglitazone | Thiazolidinediones | Non-cases - Therapy failure | 415 | 0,723 | 0,596 | 0,878 | 0,952 | 0,007 | 0,977 | 0,922 | 1,032 | 0,979 | 0,903 | 1,060 |
| Rosiglitazone | Thiazolidinediones | Cases - Therapy failure | 51 | 1,378 | 1,070 | 1,775 | 1,313 | 0,045 | 0,895 | 0,705 | 1,104 | 1,187 | 0,946 | 1,475 |
| Rosiglitazone | Thiazolidinediones | Non-cases - Therapy failure | 244 | 0,726 | 0,563 | 0,935 | 0,953 | 0,045 | 0,980 | 0,919 | 1,043 | 0,969 | 0,872 | 1,075 |
| Saxagliptin | Dipeptidyl Peptidase 4 (Dpp-4) Inhibitors | Cases - Therapy failure | 6 | 0,638 | 0,316 | 1,289 | 0,670 | 0,378 | 0,643 | 0,336 | 1,029 | 0,706 | 0,405 | 1,161 |
| Saxagliptin | Dipeptidyl Peptidase 4 (Dpp-4) Inhibitors | Non-cases - Therapy failure | 62 | 1,568 | 0,776 | 3,168 | 1,050 | 0,378 | 1,006 | 0,933 | 1,081 | 1,080 | 0,878 | 1,317 |
| Sitagliptin | Dipeptidyl Peptidase 4 (Dpp-4) Inhibitors | Cases - Therapy failure | 92 | 1,119 | 0,929 | 1,347 | 1,102 | 0,348 | 0,863 | 0,723 | 1,014 | 0,991 | 0,836 | 1,169 |
| Sitagliptin | Dipeptidyl Peptidase 4 (Dpp-4) Inhibitors | Non-cases - Therapy failure | 542 | 0,894 | 0,742 | 1,076 | 0,985 | 0,348 | 0,986 | 0,934 | 1,038 | 1,003 | 0,934 | 1,076 |
| Sulfonylureas [NOS] | Sulfonylureas | Cases - Therapy failure | 2 | 3,296 | 0,793 | 13,698 | 2,531 | 0,392 | 0,946 | 0,376 | 1,726 | 1,382 | 0,665 | 2,612 |
| Sulfonylureas [NOS] | Sulfonylureas | Non-cases - Therapy failure | 4 | 0,303 | 0,073 | 1,261 | 0,768 | 0,392 | 0,997 | 0,920 | 1,077 | 0,910 | 0,487 | 1,583 |
| Thiazolidinediones [NOS] | Thiazolidinediones | Cases - Therapy failure | 5 | 16,480 | 4,161 | 65,265 | 5,423 | 0,000 | 1,500 | 0,749 | 2,461 | 1,868 | 1,037 | 3,163 |
| Thiazolidinediones [NOS] | Thiazolidinediones | Non-cases - Therapy failure | 2 | 0,061 | 0,015 | 0,240 | 0,329 | 0,000 | 0,992 | 0,915 | 1,071 | 0,636 | 0,303 | 1,207 |
| Tolazamide | Sulfonylureas | Cases - Therapy failure | 1 | 0,824 | 0,144 | 4,717 | 0,844 | 0,757 | 1,073 | 0,371 | 2,071 | 1,239 | 0,551 | 2,484 |
| Tolazamide | Sulfonylureas | Non-cases - Therapy failure | 8 | 1,214 | 0,212 | 6,948 | 1,024 | 0,757 | 0,999 | 0,922 | 1,078 | 0,992 | 0,600 | 1,564 |
| Troglitazone | Thiazolidinediones | Cases - Therapy failure | 3 | 0,507 | 0,189 | 1,359 | 0,542 | 0,354 | 0,985 | 0,432 | 1,721 | 1,087 | 0,555 | 1,962 |
| Troglitazone | Thiazolidinediones | Non-cases - Therapy failure | 39 | 1,972 | 0,736 | 5,285 | 1,069 | 0,354 | 1,000 | 0,925 | 1,076 | 1,006 | 0,778 | 1,286 |
| Vildagliptin | Dipeptidyl Peptidase 4 (Dpp-4) Inhibitors | Cases - Therapy failure | 6 | 0,449 | 0,224 | 0,900 | 0,485 | 0,073 | 0,676 | 0,354 | 1,082 | 0,631 | 0,362 | 1,038 |
| Vildagliptin | Dipeptidyl Peptidase 4 (Dpp-4) Inhibitors | Non-cases - Therapy failure | 88 | 2,225 | 1,112 | 4,455 | 1,078 | 0,073 | 1,008 | 0,936 | 1,081 | 1,076 | 0,903 | 1,273 |
